# Selection preserves Ubiquitin Specific Protease 4 alternative exon skipping in therian mammals

**DOI:** 10.1038/srep20039

**Published:** 2016-02-02

**Authors:** Caitlyn Vlasschaert, Xuhua Xia, Douglas A. Gray

**Affiliations:** 1 Ottawa Hospital Research Institute, Ottawa, K1H 8L6, Canada; 2 Department of Biology, University of Ottawa, Ottawa, K1N 6N5, Canada; 3 Ottawa Institute of Systems Biology, Ottawa, K1H 8M5, Canada; 4 Department of Biochemistry, Microbiology and Immunology, University of Ottawa, Ottawa, K1H 8M5, Canada

## Abstract

Ubiquitin specific protease 4 (USP4) is a highly networked deubiquitinating enzyme with reported roles in cancer, innate immunity and RNA splicing. In mammals it has two dominant isoforms arising from inclusion or skipping of exon 7 (E_7_). We evaluated two plausible mechanisms for the generation of these isoforms: (A) E_7_ skipping due to a long upstream intron and (B) E_7_ skipping due to inefficient 5′ splice sites (5′SS) and/or branchpoint sites (BPS). We then assessed whether E_7_ alternative splicing is maintained by selective pressure or arose from genetic drift. Both transcript variants were generated from a USP4-E_7_ minigene construct with short flanking introns, an observation consistent with the second mechanism whereby differential splice signal strengths are the basis of E_7_ skipping. Optimization of the downstream 5′SS eliminated E_7_ skipping. Experimental validation of the correlation between 5′SS identity and exon skipping in vertebrates pinpointed the +6 site as the key splicing determinant. Therian mammals invariably display a 5′SS configuration favouring alternative splicing and the resulting isoforms have distinct subcellular localizations. We conclude that alternative splicing of mammalian USP4 is under selective maintenance and that long and short USP4 isoforms may target substrates in various cellular compartments.

Ubiquitin-specific protease 4 (USP4) is a deubiquitinating enzyme that can edit or remove ubiquitin chains of various topologies. USP4 can remove both degradative K48- and regulatory K63-linked ubiquitin chains from a growing list of protein targets that include key players in a number of signal transduction pathways. Its substrates include the transforming growth factor-*β* (TGF-*β*) I receptor^1^,^2^ and the TGF-*β*-activated kinase 1 (TAK1)^3^, Wnt/*β*-catenin pathway transcription factor Nemo-like kinase (NLK)^4^, the p53 antagonist ARF-BP1^5^, anti-viral response mediator RIG-I^6^, TNF*α*/NF-*κ*B inflammatory pathway mediators TRAF-2 & -6^7^,^8^, and the master growth factor signalling kinase PDK1^9^. Homeostatic regulation of USP4 deubiquitination targets is provided by the opposing actions of E3 ubiquitin ligase enzymes that promote degradation or complex assembly.

USP4 has both nuclear importing and exporting signal motifs and can shuttle between nucleus and cytoplasm^10^. Subcellular localization is partially controlled by AKT-mediated phosphorylation of the serine residue at position 445, which promotes cytoplasmic retention^2^. Nuclear localization, coupled with the observation that USP4 interacts with at least eleven splicing factors^11^ including Prp3^12^, suggests that USP4 regulates spliceosomal activity. This is particularly interesting since multiple spliced variants of USP4 have been reported in human. The two major isoforms differ by the inclusion of Exon 7 (E_7_; Fig. 1), though neither differential activities nor specificities have been attributed to the resulting long and short isoforms since their discovery^13^,^14^ shortly after that of the gene itself^15^. The two isoforms (with or without E_7_) both produce functional USP4 proteins in deubiquitination assays^14^. E_7_ forms the major part of the flexible linker region that enables the critical interaction of the neighbouring DUSP-UBL1 and insert domains of USP4 (Fig. 1B). In the E_7_-skipped (short) isoform, the linker is shortened from sixty-seven to twenty amino acids though retains sufficient flexibility to enable comparable domain interaction^16^. Despite the proposed facultative role of this alternatively spliced exon^16^, the majority of conserved phosphorylation sites in USP4 are within E_7_, suggesting the protein isoforms may be differently regulated^17^. What is more, the longer variant is the principal isoform and the relative amount of exon-skipped transcripts is consistent within but different among tissue types, suggesting alternative splicing may be regulated^14^. The mechanism for E_7_ skipping in human USP4 mRNA is not known, nor are the functional implications of this alternative splicing.

Exon skipping is the most frequent form of alternative splicing and accounts for about 40% of all alternative splicing in higher eukaryotes^18^,^19^,^20^. A number of exon skipping mechanisms have been proposed, including:

1. differential strength of splice signals leading to alternative splice site selection (reviewed in Keren *et al.*^21^).
2. excessively long flanking introns^22^,^23^,^24^,^25^,^26^,^27^.
3. intermixture of major and minor class introns^28^.

As USP4 does not have minor class introns, we chose to examine the first two mechanisms of E_7_ skipping, as illustrated in Fig. 1A(i,ii). First, if an exon is functionally important, then flanking sequences recognized by splicing factors will be under strong purifying selection to maintain proper spliced end-products. If the inclusion of E_7_ is unimportant, then its proximal splice sites (3′SS_6_, 5′SS_7_, and BPS_6_) may experience greater sequence drift, leading to weakening of these sites relative to 5′SS_6_, 3′SS_7_ and BPS_7_ and exon 7 skipping (Fig. 1A(i)). Selection can however also act to preserve differential splice signal strengths in order to enable the production of two isoforms. In short, strong 5′SS_6_, 3′SS_7_ and BPS_7_ relative to 5′SS_7_, 3′SS_6_ and BPS_6_, resulting from passive drift or active selection, could permit E_7_ skipping. Second, exons flanked by long introns are known to be intermittently skipped. As such, the alternative inclusion of E_7_ in USP4 mRNA could be explained by appropriately long flanking introns (Fig. 1A(ii)). We evaluated these alternative hypotheses by constructing a computational evolutionary framework to characterize USP4 splicing patterns in multiple vertebrate lineages and tested our *in silico* predictions in the laboratory to gauge whether the two isoforms of USP4 observed in humans have discrete functional and/or regulatory roles or whether they are the by-product of reduced selection for E_7_ retention.

## Results

### Bioinformatic analysis

To study exon-intron architecture and splice signal conservation, we downloaded GenBank sequences for well-annotated USP4 sequences for 62 vertebrate species covering major vertebrate taxa (see Supplementary Table S1).

#### Sequence and length conservation of USP4 exons

**Figure 1:**
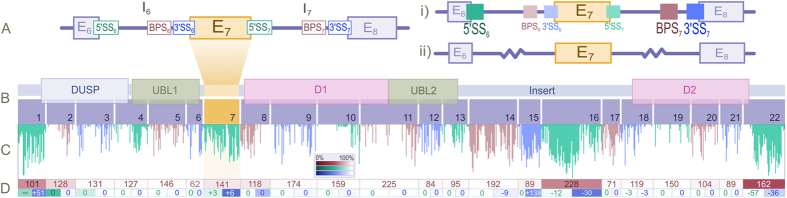
Schema of *Usp4* exonic properties and potential mechanisms of exon skipping. (**A**) Proposed alternative mechanisms for skipping of exon 7 in USP4: (**i**) differential splice site strengths (**ii**) long flanking introns (**B)** *Usp4* exon structure relative to encoded protein domains. *D1* and *D2* form the cysteine protease catalytic domain that effectuates ubiquitin cleavage. The regulatory *DUSP-UBL1* domain physically interacts with the unstructured *Insert* region and with *DUSP-UBL1* domains of other USP4 monomers leading to dimerization. (**C)** USP4 protein sequence entropy. USP4 exon-retained isoform amino acid sequences for 62 species were aligned using MUSCLE^51^. Shannon entropy was calculated using DAMBE^50^ and plotted using the ggplot2^52^ package in R. (**D)** Exon length variability. Mode of length per exon (in nucleotides) for 37 mammals indicated in pink boxes. Mode of 33 birds and 24 fish exon lengths relative to mammalian mode (codon indels) indicated in green and blue boxes, respectively. Shading of boxes indicates fraction of total sequences represented by the mode, where darkening is commensurate with increasing variability (see inset legend above).

Both long and short isoforms of USP4 comprise a bi-partite catalytic domain and a regulatory DUSP-UBL1 domain, where the seventh exon forms part of the unstructured flexible linker between these two (Fig. 1B). Sequence identity (Fig. 1C) and length (Fig. 1D) of USP4 exons are highly conserved for exons that encode structured functional domains and less well for exons that correspond to unstructured regions. E_7_ exhibits greater length variation than its neighbors. Relative to mammals, E_7_ of birds and fish generally encodes one and two additional amino acids respectively, while the length of E_7_ within each of these clades is variable; this suggests multiple indel events in USP4 during the evolutionary diversification of vertebrates. Sequence conservation among aligned USP4 exons, quantified using Shannon entropy in Fig. 1C, reveals that E_7_ is located within a highly variable region. The entropic nature of E_7_ conforms to the general pattern that alternatively spliced exons exhibit less sequence conservation^29^, which can extend to exon-intron boundaries and thus weaken the signals of proximal splice sites. However, high entropy is also observed in all other USP4 exons coding for unstructured regions (i.e., exons 1, 14, 15, 16, 17, 22), which are not alternatively spliced. The disordered nature of the unstructured region encoded by E_7_ could simply permit its sequence variability and the splice sites may not be affected. To determine whether E_7_ neighboring splice site signals are weakened, we quantified their strengths relative to the optimal sequences recognized by the spliceosome.

#### Relative strengths of branchpoint site signals

**Figure 2:**
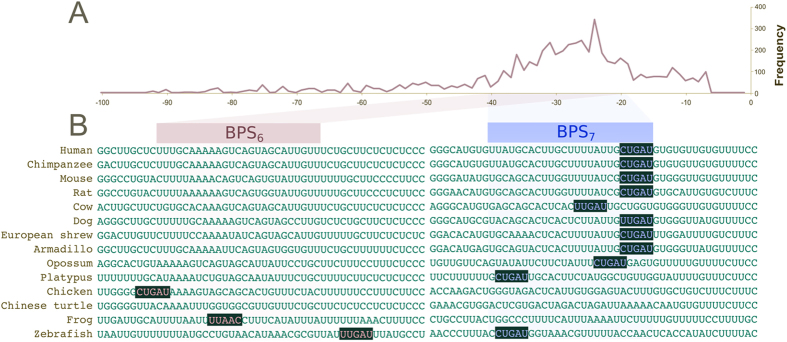
Characterization of relative BPS strengths. (**A**) Distribution of location of strong BPS sequences (“YURAY” motif with single downstream AG dinucleotide corresponding to the 3′SS) in all introns of human chromosome 22. The X-axis depicts the last 200 nt of the introns. Introns shorter than 200 nt were excluded to avoid statistical bias. D_to3′.opt_ is 20–40 nt. (**B**) Locations of YURAY sequences within 50nt of 3′ ends of introns 6 and 7 of USP4 in 14 vertebrate species.

The signal strength of a branch point site (BPS) in vertebrates is generally contingent upon the presence of a YURAY motif located at an optimal distance from 3′ end of the intron (D_to3′.opt_) followed by a pyrimidine-rich tract and the AG dinucleotide of the 3′SS signal. D_to3′.opt_ can be revealed by mapping the intronic locations of several BPS motifs and deriving an optimal range of values for these. A previous analysis of 397 human housekeeping genes reported D_to3′.opt_ to be 21–34 nt, representing 83% of introns in these highly expressed genes^30^. In Fig. 2A, we have derived a more sensitive D_to3′.opt_ of 20–40 nt based on the locations of consensus BPS sequences (YURAY) within all 15770 introns of human chromosome 22 protein-coding genes. This slightly larger window for D_to3′.opt_ will reduce the false negative rate in detecting true BPSs in the neighbouring introns of E_7_. If a strong BPS is absent from the upstream intron (I_6_), a strong downstream BPS within I_7_ could lead to exon skipping (Fig. 1A). We therefore evaluated the relative strengths of USP4 BPS_6_ and BPS_7_ for 14 well-studied species representing the major vertebrate taxa.

The location of YURAY motifs in I_7_ (Fig. 2B) suggests a strong BPS_7_ among all studied mammalian species. A strong BPS_7_ is also present in I_7_ of zebrafish (*Danio rerio*), but is missing in the frog (*Xenopus tropicalis*), the chicken (*Gallus gallus*), and the Chinese turtle (*Pelodiscus sinensis*). The latter two species retain candidate YURAY motifs which can be eliminated from consideration due to their greater distance from the 3′SS (73nt for the chicken, 85 nt for the Chinese turtle) and to the presence of a downstream non-3′SS AG (non-3′SS AG dinucleotides between a BPS and a 3′SS AG are avoided as the first AG following the branch-point is generally used as the 3′SS AG).

YURAY motifs are absent from the 40 last nucleotides of USP4-I_6_ in all mammalian species. Taken together, mammalian species in general have a weak BPS_6_ and a relatively strong BPS_7_. This lends plausibility to the first hypothesis from Fig. 1A, wherein E_7_ splicing results from alternative pairing of 5′SS_6_ and BPS_7_ (E_7_ skipping) or of 5′SS_7_ and BPS_7_ (E_7_ inclusion). In contrast, non-mammalian species tend to have a strong BPS6 (except for the Chinese turtle). YURAY motifs are located near D_to3′.opt_ in I_6_ of the chicken (D_to3′_ = 47 nt) and zebrafish (D_to3′_ = 16 nt) and frog (three YURAY motifs with D_to3′_ = 36, 42 and 53 nt, respectively). This suggests that E_7_ skipping should be less likely in non-mammalian than in mammalian species, though the Chinese turtle may be an exception.

#### Relative strengths of proximal and distal splice signals

In addition to a weak BPS_6_ relative to BPS_7_, a stronger 5′SS_6_ than 5′SS_7_ and/or stronger 3′SS_7_ than 3′SS_6_ would favor E_7_ skipping (Fig. 1A(i)). Position weight matrices (PWM) measures site-specific nucleotide usage bias in a motif alignment, where the consensus motif typically has the highest PWM score (PWMS). PWMSs are routinely used to characterize the signal strength of splice sites^31^,^32^.

A PWM for the 5′SS sequences of human chromosome 22 introns (Table 1) shows a consensus 5′SS consistent with what has been documented in the literature, i.e., a core motif of AG|GUAAGU, where “|” indicates the exon-intron junction. PWMs derived from zebrafish and chicken chromosomes are almost identical to the human 5′SS matrix in Table 1(upper panel). Thus, this PWM can be used to generate PWMSs as comparable measures of signal strength at 5′SS_6_ and 5′SS_7_ from the 14 representative vertebrate USP4 sequences, where significantly larger scores indicate stronger splice signal strength. Given Table 1(upper panel), the maximal PWMS is 16.9. PWMSs are consistently larger for 5′SS_6_ than 5′SS_7_ for the ten mammalian USP4 sequences (Table 1(lower panel)), with the mean PWMS being 9.2314 for 5′SS_6_ and 5.455 for 5′SS_7_ (t = 19.759, df = 11, p < 0.0001, paired-sample t-test). This lends support for the scenario depicted in Fig. 1A(i), where a stronger 5′SS_6_ favors E_7_ skipping in mammals. In constrast, for chicken and zebrafish, PWMS_6_ is greater than PWMS_7_ while *Pelodiscus sinensis* again conforms to the mammalian pattern (PWMS_6_ > PWMS_7_).

There were no significant differences in flanking 3′SS PWMSs (as might be expected since 5′SS and BPS are most important for determining exon-intron boundaries^33^).

#### Length of the introns flanking exon 7

**Figure 3:**
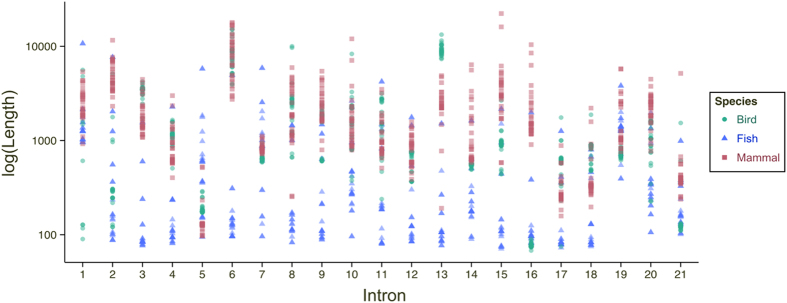
Length of USP4 introns for 62 bird, fish and mammal species. log10 scale used for y-axis. Intron 6 is highly variable in length.

Because exons flanked by long introns tend to be skipped during the splicing process^22^,^23^,^24^,^25^,^26^,^27^, we have examined whether E_7_ is flanked by long introns. As observed in Fig. 3, I_6_ varies dramatically in length between clades. The average length of I_6_ is the largest amongst all introns for mammals ([2733, 17911] nt) and second largest for birds ([3903, 15190] nt; second to I_13_). Whereas I_6_ is also relatively long in cartilaginous fish, the earliest-diverging clade presenting USP4 (shark = 4961 nt), it is contrastingly short in bony fish ([96, 310] nt). Altogether this suggests a large number of indels during the evolution of different vertebrate lineages, likely the most amongst all introns of USP4. Although exons with long flanking introns tend to be lost in the final mRNA^22^,^23^,^24^,^25^,^26^,^27^, there is discrepancy concerning the relative effect of upstream and downstream introns. The detailed experimental study on CD44^22^ shows that exon skipping occurs only when the exon is flanked on both sides by long introns, and that the effect of the two introns appears to be symmetrical. However, subsequent studies on the relationship between intron length and exon skipping^23^,^24^ are not always consistent with these earlier findings. In particular, it appears as though the upstream intron has a greater effect on exon skipping than the downstream intron^25^, which would suggest that the the potential contribution of the long I_6_ to E_7_ skipping in mammals should not be ignored.

### Experimental tests of alternative hypotheses

#### Determination of splicing mechanism

**Figure 4:**
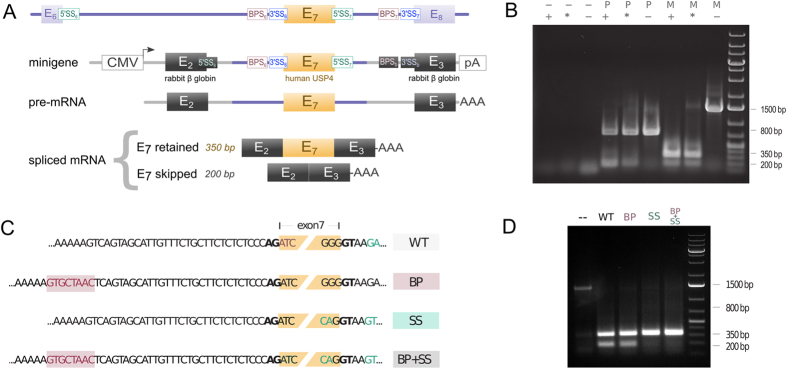
Mismatched flanking 5′ splice site strengths leads to alternative skipping of exon 7. (**A**) Schematic of the RT-PCR assay construct. Assays were performed using oligonucleotide primers positioned upstream and downstream of the rabbit exons. (**B**) Exon skipping as detected in minigene RT-PCR assay. Lane labels specify transfected construct and reagents in U2OS cells. First line: −: untransfected control, ***P***: empty plasmid (pXJ41), ***M***: USP4-E7 minigene construct (pDG467). Second line: +: oligo dT-primed cDNA reaction, *: random-primed cDNA reaction, −: random primers, no reverse transcriptase (RT). A 200 base pair (bp) product of is visible after primed reactions of pXJ41-transfected cells. Exon-retained and -skipped products are visible as 350 and 200 bp bands, respectively, in pDG467-transfected cells. Products of approximately 800 and 1500 bp were generated pXJ41- and pDG467-transfected conditions, respectively, in the absence of RT and likely arose from amplification of DNA rather than cDNA. (**C**) Schematic of site-directed mutations. The wild type (WT) sequences and the boundaries of E_7_ (yellow box) are shown. In the branch point (BP) mutant a YURAY sequence (indicated in pink) was inserted 32 residues upstream of E_7_. In the splice site (SS) mutants the downstream exon-intron boundary sequence were optimized (substituted residues indicated in green). The combination mutant (BP + SS) incorporated both mutations. (**D**) RT-PCR analysis of site-directed mutants. Lane labels correspond to transfected mutant constructs as per Figure 4C. Exon-skipped products are absent from SS and BP + SS mutants.

Although our results are consistent with the hypothesis that E_7_ skipping results from BPS_7_ strengthening relative to BPS_6_ and 5′SS_6_ stronger than 5′SS_7_, they do not exclude the possibility that the longer intron I_6_ or other potentially complicating factors in USP4 pre-mRNA may contribute to E_7_ skipping. To test the hypothesis that E_7_ skipping is due to differential splice signals at 5′SS and BPS, we created a minigene construct by inserting the human USP4 genomic sequence encompassing E_7_ (together with 75 nt at the 3′ tail of I_6_ and 51 nt at the 5′ end of I_7_) into the well characterized splicing reporter pXJ41 (the generous gift of Dr. Sushma Grellsheid, Durham University). In the resulting minigene the human E_7_ genomic fragment resides in the second intron of the rabbit beta hemoglobin gene (Fig. 4A). If E_7_ skipping is due to the long upstream I_6_, then we should observe no E_7_ skipping in this construct with short upstream intron. The minigene mimics the scenario in Fig. 1A(i) with the differential signal strength of splice sites: 5′SS_a_ (Fig. 4A) is stronger (PWMS = 7.5358) than 5′SS_7_ (PWMS = 5.0898) and BPS_b_ (Fig. 4A) is stronger than BPS6, with the former having a CUAAC (YURAY) sequence located 35 nt from the 3′ end of the intron and the latter having no YURAY at D_to3′.opt_. If E_7_ skipping in its natural USP4 mRNA is due to such differential strength of splice signals, then we should observe E_7_ skipping in the minigene mRNA.

When the minigene was expressed in human U2OS osteosarcoma cells, RT-PCR analysis using primers upstream and downstream of the rabbit exons revealed two isoforms of the size predicted for retention and exclusion of E_7_ (Fig. 4B). Similar results were obtained in the unrelated HeLa cell line (not shown). This finding excludes exon skipping as a consequence of intron length but is consistent with E_7_ skipping due to the differential strength of splice signals. We therefore explored the contributions of the BPS_6_ and 5′SS_7_ elements by site-directed mutagenesis of the E_7_ minigene construct, inserting a consensus YURAY sequence at D_to3′.opt_ in I_a_ (upstream of E_7_) and/or engineering an optimized 5′SS_7_ element in I_b_ (depicted as BP and SS respectively in Fig. 4C). The mutated versions of the minigene were transfected into U2OS cells and RT-PCR analysis was performed as before. Whereas the introduction of the consensus YURAY sequence had no effect on the ratio of exon retained and exon excluded products, the latter was undetectable in RNA isolated from cells transfected with minigenes in which the 5′SS_7_ element had been optimized (Fig. 4D).

#### Phylogenetic distribution of USP4 alternative splicing

**Figure 5:**
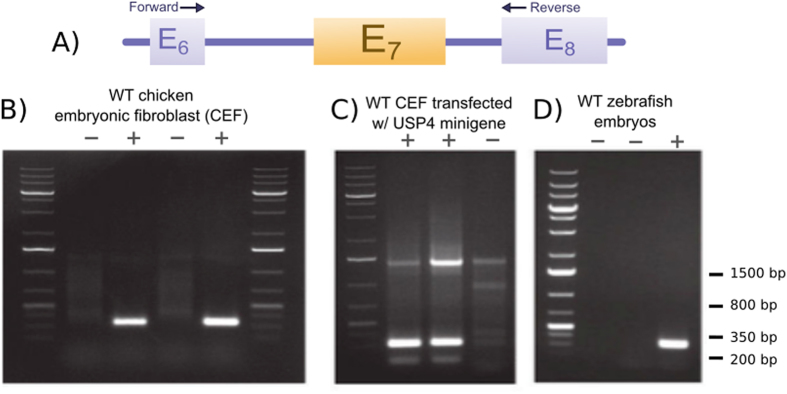
Exon skipping does not occur in the USP4 gene of the chicken or the zebrafish. (**A**) Schematic of the RT-PCR strategy using primers specific for exons 6 and 8. (**B**) RT-PCR analysis of RNA from the endogenous USP4 gene in chicken. Lane labels specify addition (+) or omission (−) of RT (control) to chicken embryo fibroblast (CEF) RNA extracts. When RT was included a single PCR product of predicted size (329 bp) for the exon-retained transcript was detected. (**C**) RT-PCR analysis of CEF transfected with the human USP4 minigene construct. Lane labels specify transfection of pDG467 (+) or an irrelevant control plasmid (−) in CEF cells. Both the exon-retained and exon-included products were detected (as in human cells). (**D**) RT-PCR analysis of RNA from the endogenous USP4 gene in the zebrafish. Lane labels specify addition (+) or omission (−) of template (lane 2) or RT (lane 3) to larval zebrafish RNA extracts. A single amplification product was detected of the size predicted for the exon-retained cDNA (313 bp).

In contrast to mammalian species, 5′SS_6_s of chicken and zebrafish USP4 are weaker than 5′SS_7_s, as indicated by their PWMS values (Table 1). To verify whether (as would be predicted) E_7_ skipping does not occur in such species, RT-PCR analysis was performed on RNA isolated from primary chick fibroblast cultures (the generous gift of Dr. J. S Diallo, Ottawa Hospital Research Institute). Primers corresponding to sequences in exons 6 and 8 were used to detect the presence or absence of the seventh exon as depicted in Fig. 5A. We detected only the exon-retained version of the transcript (Fig. 5B). However, when the human minigene was introduced into chick embryo fibroblasts by transfection both isoforms were detected (Fig. 5C). The absence of exon skipping in the chicken cells could thus be directly attributed to the primary sequence of the chicken USP4 pre-mRNA. Our data exclude the possibility that E_7_ retention occurs in the chicken as a consequence of an altered repertoire of splicing factors in avian versus mammalian cells (see Discussion). By similar logic we predict that exon skipping would not occur in the zebrafish gene; RT-PCR analysis of RNA from larval stage zebrafish (the generous gift of Dr. Marc Ekker, University of Ottawa) confirmed the presence of a single exon-retained isoform (Fig. 5D). In support of this, performing a BLASTn of USP4 exons 5–13 against the 600,432 chicken EST sequences recovered four sequences with E_7_ but no sequence without E_7_. Among the 1,488,339 zebrafish ESTs, seven have E_7_ but none are without E_7_. In contrast, searching the 8,704,868 human ESTs recovered five sequences with and seven without E_7_. The corresponding numbers from the 4,853,570 mouse EST sequences are 20 and 8, respectively. Our conceptual framework based on the relative strengths of 5′ splice signals thus correctly predicted splicing propensity in these model organisms, confirmed by both database and experimental analyses.

As is shown in Fig. 4D, the optimization of three nucleotides in the 5′ splice site downstream of USP4-E_7_ according to the consensus sequence, namely −3G → C, −2G → A and +6A → T, proved sufficient to eliminate exon skipping in the human USP4 minigene. Among species observed in Table 1, the nine therian mammal 5′SS_6_s feature optimal nucleotides −2A and +6T while suboptimal −3G, −2G and +6A penalize the 5′SS_7_ PWMSs of all members of this lineage. These residues are identical in the Chinese turtle and are likely thus responsible for the observed alternative E_7_ skipping in this distant relative. In contrast, both flanking splice sites of E_7_ in zebrafish feature optimal nucleotides (5′SS6: −3A, −2A, +6T; 5′SS7: −3C, −2A, +6C), which preclude E_7_ exclusion. Curiously, in chicken, these determinant nucleotides are identical to those of mammals which produce E_7_ skipping with the exception of the 5′SS_6_ +6N site, which is weak (+6A). The upstream and downstream 5′SSs in chicken, though weak, are equivalent and prevent exon skipping as in zebrafish. The +6N site may thus be the discriminant factor in E_7_ skipping propensity. To verify this, we expanded the scope of our analysis to include all sequenced genomes bearing USP4 to see whether the 5′SS mismatching (in particular +6 site mismatching) predicts splicing proclivity. While direct expansion of our analytical framework is limited by insufficient EST data and biological sample unavailability, we can infer splicing patterns from RNA-seq datasets. Similar to the methodology used for EST mining, we performed a BLASTn of available RNA-seq data from the Sequence Read Archive (SRA) using the USP4 coding sequence with E_7_ removed as a query. In the absence of hits crossing the exon 6–8 boundary for multiple, sufficiently large expression datasets, species were deemed to forgo short isoform production.

**Figure 6:**
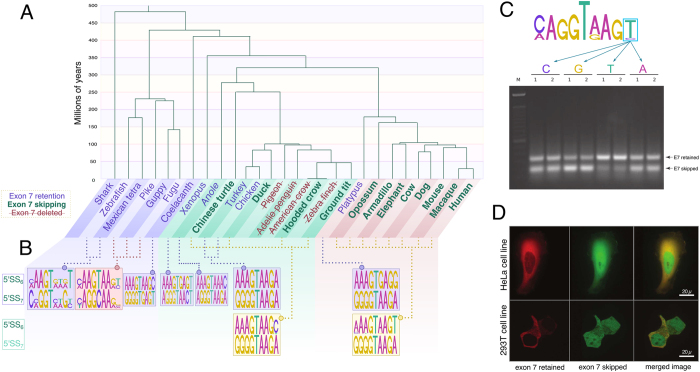
Long and short isoform production was selected for in therian mammals. (**A**) USP4 exon 7 skipping propensity throughout the vertebrate phylogeny (see inset Legend). (**B**) 5′SS_6_ and 5′SS_7_ identities for species in (**A**). In (**B**), splice site configurations that lead to constitutive retention of E_7_ are in the top row and those that lead to alternative skipping are in the bottom row. (**C**) Sequence logo for an optimal 5′SS (from Table 1) and changes in long-to-short isoform ratios (IRL/S) after experimental replacement of the sixth intronic nucleotide (+6 site) of the downstream 5′SS_7_ of human USP4 in H1299 cells. IRL/S quantifications are as follows: C = 1.00, G = 0.81, T = 1.52, A (WT) = 1.02. (**D**) Subcellular localization of exon 7 skipped and exon 7 retained USP4 isoforms. Long and short isoforms with appended green (GFP) and red (mKATE) fluorescent tags, respectively, were transfected into 293T and HeLA cells.

Figure 6A summarizes USP4 splicing patterns in a phylogenetic context with corresponding flanking 5′SS sequence logos indicated. According to the PWM in Table 1A, +6A and +6G weaken the 5′SS while +6T is optimal and +6C is neutral (weighted consensus illustrated in Fig. 6C). For all tetrapods, when the downstream +6 site is stronger than the upstream +6 site, there is alternative splicing of E_7_. This correlation is particularly apparent in the avian phylum: chicken and turkey have no E_7_ skipping (+6A; +6A), all other birds either exhibit skipping (+6C/T; +6A) or loss of E_7_. What is more, some members of sister taxa have lost the ability to produce the long isoform: E_7_ is deleted in *Corvus brachyrhynchos* but present in *Corvus cornix cornix*; absent from Adelie penguin but present in Emperor penguin, for example. In contrast to this substantial variability, all mammals retain an optimal E_7_ skipping configuration, +6T; +6A (with the exception of the clade root: platypus USP4 has +6G; +6A and, consistent with our model, does not undergo skipping). In theory, many nucleotides substitutions could disrupt the 5′SS if alternative splicing were the result of drift. Since the same splice site configuration is maintained throughout 220 million years of mammalian evolution there may be selection for this particular +6 configuration. In Fig. 6C and Supplemental Figure 1, we show the effects of downstream +6 site point mutation from native +6A to +6T, +6C and +6G in human and mouse cell lines. In each case, the splicing propensity changed in direct relation with the estimated fitness in our PWM: alternative splicing was nearly eliminated in +6T, slightly reduced in +6C and increased in +6G. Therefore, we propose that the highly conserved +6A site within 5′SS_7_ is under natural selection to maintain both long and short USP4 isoforms in therian mammals.

#### Differential localizations and roles of spliced isoforms

The evidence supporting alternative splicing selection in mammalian USP4 is strong; we would consequently expect the two isoforms to have distinct cellular roles. Indeed, we observed distinct subcellular localizations of long and short USP4 isoforms in both single- and double-transfections of HeLa, U2OS, 293T and 3T3 cells (see Fig. 6D). While the short isoform was distributed throughout the cell, the localization of the long isoform was largely cytoplasmic in most if not all cells in the four cell lines examined. Potential implications of this observation are discussed below. Altogether, our results suggest that the two major USP4 isoforms generated by alternative skipping of its seventh exon may not be functionally redundant as previously suggested.

## Discussion

It has been proposed that mutations that weaken the 5′ splice site are responsible for the evolutionary shift from constitutive to alternative splicing in many vertebrate genes, as reviewed in Keren *et al.*^21^, and compelling evidence has been presented in support of this hypothesis^34^. While most minor splice variants are attributable to noisy splicing^35^,^36^, USP4 constitutes a rare case wherein selective pressure acts to conserve differential 5′SS strengths leading to exon skipping in therian mammals. The approach we presented here focuses on these cis-acting splice sites, which offers a more basic but more direct framework towards understanding the splicing code. 5′ splice sites can recruit trans-acting alternative splicing factors for intrinsic splice regulation. For example, deleterious exon skipping in survival of motor neuron (SMN) pre-mRNA can be attributed to recruitment of splice repressor U2AF65 by a weak downstream 5′SS^37^. While trans-acting factors interacting with the 5′SSs of USP4 may similarly regulate E_7_ skipping, our model explains USP4-E_7_ splicing propensity independent of other cis-regulatory sequences such as exonic splice enhancers (ESEs), which may or may not be selectively co-optimized in USP4 alternative splicing. Our study also highlights the importance of experimental verification of alternative hypotheses. Although the bioinformatics framework alone cannot distinguish between the two mechanisms proposed in Fig. 1(i,ii), the experimental results demonstrate that relative 5′SS strengths are far better predictors of alternative splicing than BPSs or upstream intron lengths. Further, our combinatorial *in silico* and experimental approach identified the +6 site within the 5′SS as the splicing discriminant. Intronic +6 site mutations have been reported as splicing instigators in other genes such as SMN1^38^,^39^. E_7_ skipping in SMN1 leads to spinal muscular atrophy (SMA), and SNPs that cause this aberrant skipping have been identified in patients at the downstream 5′SS_7_ at the +6 site (+6T → G). SMN1 has a very close paralog, SMN2, that is incapable of rescuing SMN1 deficiency in SMA because its E_7_ is also skipped due to a WT nucleotide variant, 5′SS_7_ +6G. Thus, +6T at the downstream 5′SS_7_ of SMN1/2 promotes upstream exon inclusion while +6G promotes near-total upstream exon skipping. In mammalian WT USP4, 5′SS_6_ has +6T while 5′SS_7_ has +6A. As reflected in the PWM in Table 1 and observed in Fig. 6C, the strengths of +6 site nucleotides are predicted to be as follows: T > C ≥ A > G, where a stronger 5′SS_6_ +6 relative to 5′SS_7_ +6 correlates with splicing proclivity. It is curious that +6A ≠ +6G and that the former was selected as the weak downstream nucleotide of USP4. A plausible mechanism for +6A-dependent alternative skipping may involve U1C, a component of the U1 snRNP that preferentially recognizes a 5′SS motif with +6A, GTATAA^40^, and can interact with splicing regulator TIA-1^41^,^42^ to promote exon retention, for example in SMN2^43^,^44^. Several genes undergo U1C-dependent alternative splicing^45^,^46^. Based on linear changes in the relative abundances of the short and long isoforms observed during differentiation of P19 embryonic carcinoma cells (Gray, unpublished) we postulate that E_7_ of USP4 may be subject to regulated alternative splicing in therian mammals.

Retention or exclusion of the amino acids encoded by exon 7 does not affect the protease activity of the USP4 enzyme (using a synthetic substrate^14^) and the ubiquitin-exchange regulatory mechanism proceeds equally in both USP4 isoforms^16^. We have nonetheless shown that there is selection for alternative splicing maintenance in mammalian USP4. Establishing the molecular selection driver should be highly informative. We show that the two isoforms display distinct subcellular localizations, which suggests that (1) propensity and/or (2) capacity for substrate interactions may differ. First, vital cytoplasmic (e.g. TGF-*β* pathway^1^) and nuclear (e.g. spliceosomal^11^,^12^) substrates have been reported for USP4 (isoforms specificities not declared). Long and short USP4 isoform production may be advantageous for simultaneous, collective targeting of key substrates in various cellular compartments. On the other hand, the two isoforms almost certainly have some distinct interactors. Cytoplasmic retention of USP4-long is mediated by phosphorylation of a ubiquitously conserved serine (S445); the apparent absence of this regulation in USP4-short may reflect a lack of phosphorylation by Akt^2^. Exon 7 of mammalian USP4 is serine-rich (16 out of 47 residues; human sequence: RSSTAP**S**RNFTT**S**PKSSA**S**P**Y**SSVSASLIANGDSTSTCGMHSSGVSRG) and also contains six constitutively charged residues (five positively charged and one negatively charged). While the +4 net charge difference between isoforms likely affects substrate interactions, the serine-rich exon also retains multiple phosphorylation sites (underlined) that are conserved among all mammals. USP4-E_7_ may be phosphorylated in conjunction with Ser445 for nuclear exclusion or may be required for interaction with Akt and other substrates. For instance, SART3, a spliceosomal factor and deubiquitination target of both USP4^12^ and its paralog USP15^47^, has been reported to interact with serine-rich (not to be confused with serine/arginine-rich) domains of proteins^48^. Interestingly, USP15 contains an analogous, serine-enriched alternatively spliced seventh exon (**S**PGA**S**NF**ST**LPKI**S**P**S**SLSNNYNNMNNR; reported phosphorylation sites underlined). Splice boundaries and amino acid sequence differ between E_7_ of USP4 and USP15, suggesting that alternative splicing arose independently in these, though they both maintain significant proportions of serines. This may be a case of stabilizing selection acting on clusters of phosphorylation sites^49^. There may be an important feedback loop involving the splicing and subsequent localization of USP4 and USP15, two DUBs that critically interact with the spliceosome. Long and short USP4 production and thus DUB modification of isoform-specific substrates may differ across tissue types. It remains to be seen whether such isoform-specific substrates drove evolutionary conservation of the dual isoforms within placental mammals.

To summarize, we have shown that the long and short isoforms of USP4 have distinct properties and their contributions to cellular networks should be considered separately. Most proteins have more than one reported isoform, and though most may be considered non-essential noise, distinct functional variants, such as in USP4, must not be grouped as one protein. The roles of all significantly expressed minor splice variants should be studied more carefully.

## Methods

### Bioinformatic analysis

Well-annotated USP4 sequences for 62 vertebrate species were downloaded from GenBank (See Supplementary Table S2) covering major vertebrate taxa. Coding sequences, exons, introns, and exon-intron junctions (5 nt on the exon side and 12 nt on the intron side) were extracted and analyzed by using DAMBE^50^. Shannon entropy (H) is used as a measure of site-specific sequence variability over a sliding window in Fig. 1C.


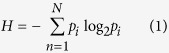


N in Eq. (1) is the number of character states (N = 20 for protein), and pi is the frequency of each amino acid. To identify the optimal distance (D_to3′.opt_) of branchpoint sites (BPS), we searched for the presence of YURAY motifs without downstream non-3′SS AG dinucleotides in all 15770 introns in human chromosome 22 and compiled D_to3′_ defined as the number of nucleotides from the beginning of YURAY (human) to the 3′ end of the intron. The range that contains the majority of D_to3′_ values is labeled D_to3′.opt_. We applied the same approach for chicken and zebrafish introns. To identify whether USP4 is subject to alternative splicing in diverse species, BLAST searches were performed using putative species-specific short isoforms (i.e., with exon 7 deleted) as a query against RNA-seq datasets from the Sequence Read Archive (SRA). RNA-Seq dataset identifiers are listed in the Supplementary Table S2.

### RT-PCR analysis of spliced isoforms

The USP4 exon 7 reporter plasmid pDG467 was generated by insertion of a segment of human USP4 genomic DNA into the minigene reporter pXJ41. A segment of the USP4 gene on chromosome 3 (49348797–49349237) was amplified by polymerase chain reaction from H1299 cell DNA using Phusion high fidelity DNA polymerase (Thermo Scientific, Waltham, MA) with the forward primer AAAAAAGAATTCATTACAGGCACGAGCCACTG and the reverse primer AAAAAAGAATTCGCCCATCCCTTCATAAACAA (annealing temperature 55C). The resulting 699 base pair DNA product was digested with EcoRI and gel purified prior to ligation into the MfeI site of pXJ41. The branch point insertion plasmid pDG484 was generated using the Phusion site directed mutagenesis system (Thermo Scientific, Waltham, MA) with pDG467 as the template, forward primer 5′-GCTAACTCAGTAGCATTGTTTCTGCTTCTC-3′, and reverse primer 5′-ACTTTTTGCAAAGAGCAAGCCCTATTTA-3′ (68C annealing temperature). The splice donor optimization plasmid pDG485 was generated with pDG467 as the template, forward primer GTAAGTGCAGGTCCTTTCACTCTGCTTC, and reverse primer CTGCTGCTGACACCGGAACTGT (68C annealing temperature). The combination plasmid pDG468 was generated using the same primers and conditions, but using pDG484 as the template. Substitutions at the +6 position of the splice site were generated by polymerase chain reaction with pDG467 as the template. For all such substitutions the reverse primer CCCCTGCTGACACCGGAACTGT was used. For the A to C substitution the forward primer was GTAAGCGCAGGTCCTTTCACTCTGCTTC. For the A to G substitution the forward primer was GTAAGGGCAGGTCCTTTCACTCTGCTTC. For the A to T substitution the forward primer was GTAAGTGCAGGTCCTTTCACTCTGCTTC. The conditions for polymerase chain reaction were as above, but with an annealing temperature of 72C. The engineered mutations in all USP4-derived plasmids were verified before subsequent transfection experiments were performed. For analysis of splicing isoforms human U2OS cells, mouse NIH 3T3 cells or human H1299 cells (ATTC, Manassas, VA) were transfected at 50% confluence in 6 well dishes with 1 μg of plasmid at 3 μl of GeneJuice (EMD Millipore, Billerca, MA) using the manufacturer’s protocol. RNA was harvested 24 hours post-transfection using a GeneJET RNA purification system (Thermo Scientific, Waltham, MA). Coupled reverse transcription/polymerase chain reaction was performed for each sample using the MyTaq One Step RT-PCR kit (BioLine, Taunton, MA) with forward primer GCTCCGGATCGATCCTGAGAACT and reverse primer GCTGCAATAAACAAGTTCTGC (60C annealing). RT-PCR products were analyzed on 1.2% agarose gels. DNA products were stained with Safe-Red (Applied Biological Materials, Inc., Richmond BC) and visualized using a UV gel camera apparatus (UVP, Upland CA).

### Microscopy and imaging

To establish the localization of exon-retained and exon skipped isoforms of USP4, cDNAs corresponding to the mouse isoforms were obtained from Origene Technologies Inc. (Rockville, MD, USA) as C-terminal fusions with the red fluorescent protein mKATE or monomeric GFP, respectively. One microgram of each plasmid was introduced into cells cultured on cover slips using the GeneJuice transfection reaction (Millipore Canada, Etobicoke ON) following the supplied protocol. 24 hours post-transfection the cells were fixed for 10 minutes in 0.4% paraformaldehyde and were mounted in Vectashield mounting media (Vector Laboratories Canada, Burlington ON). Images were acquired using a Zeiss Axiovert 200M microscope equipped with the Apotome optical sectioning module.

## Additional Information

**How to cite this article**: Vlasschaert, C. *et al.* Selection preserves Ubiquitin Specific Protease 4 alternative exon skipping in therian mammals. *Sci. Rep.* **6**, 20039; doi: 10.1038/srep20039 (2016).

## Supplementary Material

Supplementary Information

## Figures and Tables

**Table 1:** 

Site	−5	−4		−3	−2	−1	1		2	3		4	5	6	7		8
A	0.2082	0.1306		0.3720	**1.4204**	−1.5830	−8.3696		−8.9985	**1.0932**		**1.4771**	−1.9859	−0.7113	0.0161		−0.5293
C	0.5104	0.6957		**1.0657**	−0.9485	−2.3528	−10.4401		−5.2125	−2.4697		−1.0419	−1.4937	0.0111	0.3350		0.6644
G	−0.7793	−0.4697		−0.9095	−1.4947	**1.2021**	**1.4713**		−9.4051	0.3050		−1.2529	**1.1711**	−0.6257	−0.0225		−0.3267
U	0.2021	−0.3378		−1.1008	−0.5682	−1.9475	−10.5499		**2.2380**	−3.5486		−1.0776	−1.8007	**1.0212**	−0.3701		0.2039
				
**Species**			**5′SS**_**6**_					**PWMS**_**6**_			**5′SS**_**7**_					**PWMS**_**7**_	
Human			UCAAA|GUAAGUGA					8.6625			AGGGG|GUAAGAGC					5.0898	
Chimpanzee			UCAAA|GUAAGUCA					9.0200			AGGGG|GUAAGAGC					5.0898	
Mouse			UCAAA|GUAAGUGA					9.5187			AGGGG|GUAAGAAC					5.5747	
Rat			UCAAA|GUAAGUGA					9.3740			AGGGG|GUAAGAAC					5.5747	
Cow			UCAAA|GUAAGUGA					9.1264			AGGGG|GUAAGAGC					5.0898	
Dog			UCAAA|GUAAGUGA					9.1264			AGGGG|GUAAGAGC					5.5361	
Shrew			ACAAA|GUAAGUGG					9.3351			CGGGG|GUAAGCGC					6.5608	
Armadillo			CCAAA|GUAAGUCA					9.7922			AGGGG|GUAAGAGC					5.5361	
Opossum			UCCAA|GUAAGUGA					9.9648			AGGGG|GUAAGAGC					4.6637	
Platypus			UCAAA|GUAAGGGA					8.3157			CGGGG|GUAAGAGC					4.8212	
Chicken			GCAAA|GUAAGAGU					5.6036			AGGGG|GUAAGAGC					5.9924	
Zebrafish			UCAAA|GUAUGUCC					7.9026			AACAG|GUCAGCUU					8.1978	
Turtle			ACAAA|GUAAGCAU					10.6407			GGGGG|GUAAGACC					3.8889	
Frog			ACAAA|GUGAGUAU					8.5266			AAAGG|GUAACUAU					6.4370	

The upper panel provides position weight matrix (PWM) for 15770 exon-intron junctions (5’SS) from human chromosome 22. The consensus sequence (CAGjGUAAGU) is shown in bold. The five exonic nucleotides are labeled as -5 to -1 and the 8 intronic nucleotides as 1 to 8. The lower panel gives position weight matrix scores (PWMS_6_ and PWMS_7_, respectively) for the 5’ splice sites of introns 6 and 7 (5′SS_6_ and 5′SS_7_ in Fig. 1A(i)) from 14 vertebrate species according to the scoring matrix in the upper panel.
