# Supplementary material for: Selection preserves Ubiquitin Specific Protease 4 alternative exon skipping in therian mammals
Source: Sci Rep. 2016 Feb 2;6:20039. doi: 10.1038/srep20039 (PMC4735762; doi:10.1038/srep20039)
Supplement: Supplementary Information [file srep20039-s1.pdf]

Supplementary information

Selection preserves Ubiquitin Specific Protease 4 alternative exon skipping in therian mammals

Caitlyn Vlasschaert[1,3,4], Xuhua Xia[2,3], and Douglas A. Gray[1,4]

[1] Ottawa Hospital Research Institute, Ottawa, K1H 8L6, Canada
[2] Department of Biology, University of Ottawa, Ottawa, K1N 6N5, Canada
[3] Ottawa Institute of Systems Biology, University of Ottawa, Ottawa,K1H 8M5, Canada
[4] Department of Biochemistry, Microbiology and Immunology, University of Ottawa, Ottawa, K1H 8M5, Canada

Supplementary Table S1 - **USP4 GenBank accession codes**

Species	Gene ID
Anas platyrhynchos	101798997
Anolis carolinensis	100560633
Apaloderma vittatum	104273102
Apternodytes forsteri	103903383
Astyanax mexicanus	103035454
Balaenoptera acutorostrata scammoni	103020735
Balearica regulorum gibbericeps	104632144
Bos taurus	508042
Buceros rhinoceros silvestris	104489223
Callorhinchus milii	103176635
Calypte anna	103533244
Camelus ferus	102508645
Canis lupus familiaris	476624
Cariama cristata	104160674
Cavia porcellus	100728835
Ceratotherium simum simum	101400921
Charadrius vociferus	104282972
Chinchilla lanigera	102007934
Chrysochloris asiatica	102822016
Colius striatus	104558238
Columba livia	102096446
Condylura cristata	103750191
Corvus brachyrhynchos	103614391
Corvus cornix cornix	104683458
Cynoglossus semilaevis	103385916
Danio rerio	449927
Dasypus novemcinctus	101416070
Echinops telfairi	101658301
Egretta garzetta	104126658
Equus caballus	100053443
Esox lucius	105017171
Eurypyga helias	104507057
Felis catus	101083327
Fulmarus glacialis	104075120
Gallus gallus	415937
Geospiza fortis	102036086
Haplochromis burtonii	102312056
Homo sapiens	7375
Larimichthys crocea	104930487
Latimeria chalumnae	102362393
Lepisosteus oculatus	102697090
Leptosomus discolor	104354097
Loxodonta africana	100653739
Macaca mulatta	706930
Manacus vitellinus	103755327
Maylandia zebra	101487040
Meleagris gallopavo	100546721
Melopsittacus undulatus	101871505
Mesitornis unicolor	104547449
Monodelphis domestica	100021067
Mus musculus	22258
Myotis davidii	102767044
Myotis lucifugus	102422860
Neolamprologus brichardii	102796025
Nipponia nippon	104008720
Notothenia coriiceps	104957993
Ochotona princeps	101524860
Odobenus rosmarus divergens	101382527
Oreochromis niloticus	100694486
Ornithorhynchus anatinus	100073617
Oryzias latipes	101173051
Otolemur garnettii	100945313
Pan paniscus	100967331
Pan troglodytes	460367
Pelecanus crispus	104034726
Pelodiscus sinensis	102463622
Phalacrocorax carbo	104047918
Physeter catodon	102984400
Poecilia formosa	103150708
Poecilia reticulata	103465224
Pseudopodeces humilis	102114050
Pundamilia nyererei	102206398
Pygoscelis adeliae	103925502
Python bivittatus	103048751
Rattus norvegicus	290864
Sarcophilus harrisii	100933028
Serinus canaria	103816987
Sorex araneus	101544719
Stegastes partitus	103358734
Struthio camelus australis	104142900
Sus scrofa	100623171
Taeniopygia guttata	100228772
Takifugu rubripes	101079470
Tarsius syrichta	103249290
Tinamus guttatus	104576715
Vicugna pacos	102533688
Xenopus tropicalis	100491110
Xiphophorus maculatus	102224630
Zonotrichia albicollis	102067363

Supplementary Table S2 - **RNA-Seq dataset accession IDs**

Species	Dataset ID
Mexican tetra	SRX685289
Pike	SRX514270
Amazon molly	SRX208992
Fugu	SRX371979
Coelacanth	DRX001725
Xenopus	SRX1099270
Green anole	SRX111310
Chinese turtle	DRX001541
Turkey	SRX566381
Chicken	ERX524892, SRX1020699, DRX001555, ERX620932
Mallard duck	SRX255765, ERX480414, ERX554399, SRX952159
Hooded crow	SRX974016
Ground tit	SRX246871
Platypus	SRX122685
Opossum	SRX877987
Armadillo	SRX544837
Elephant	SRX386099
Cow	SRX1092528
Dog	SRX889808
Macaque	SRX858064

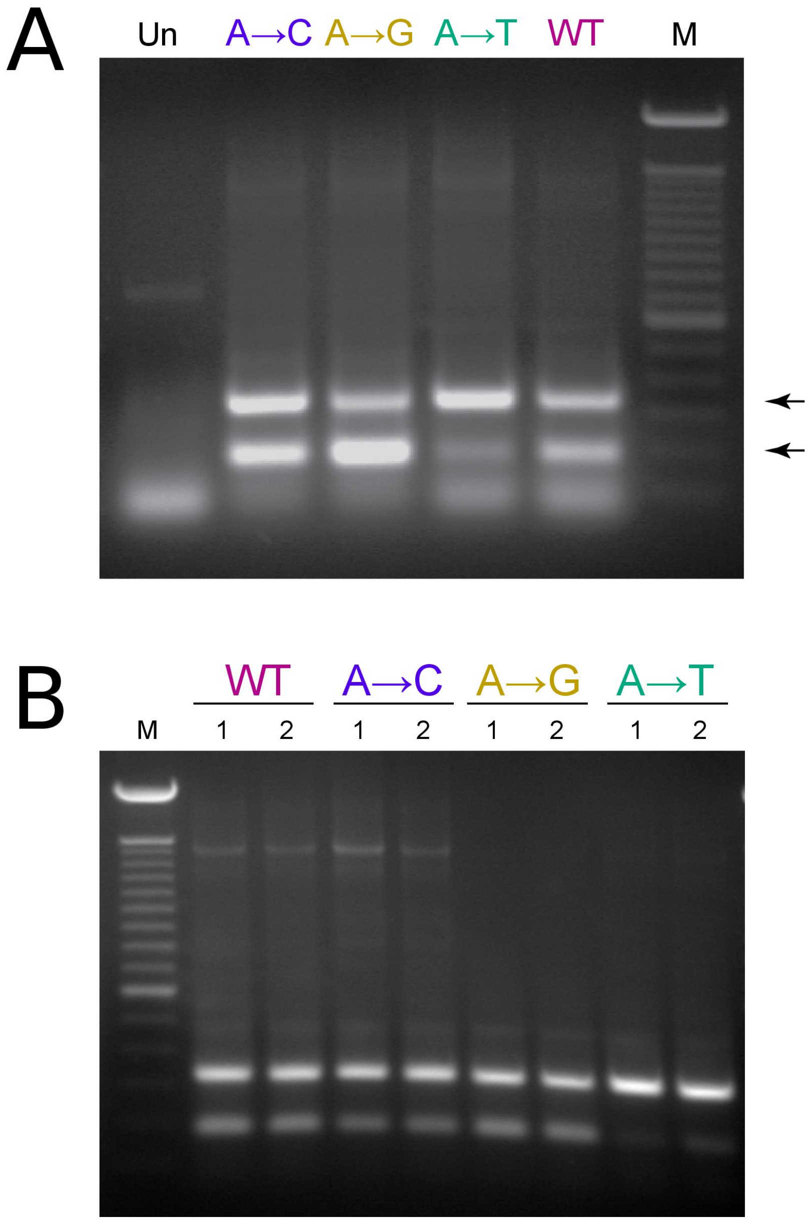

Supplementary Figure 1. Changes in long-to-short isoform ratios after experimental replacement of the sixth intronic nucleotide (+6 site) of the downstream splice site (5'SS_7) of mouse USP4 in A) NIH 3T3 (mouse embryonic fibroblasts) and B) U2OS (human osteosarcoma) cells. The wild-type (WT) +6 site is adenine (A). In both cases, replacement of A with cytosine (C) increases exon retention and replacement with thymine (T) produces almost exclusive exon retention, while replacement with guanine (G) increases exon skipping.
